# Supplementary material for: Evaluation of the Impact of Cold Atmospheric Pressure Plasma on Soybean Seed Germination
Source: Plants (Basel). 2021 Jan 19;10(1):177. doi: 10.3390/plants10010177 (PMC7833387; doi:10.3390/plants10010177)
Supplement: Supplementary file 1 [file plants-10-00177-s001.zip › Supplementary Materials Figure S1 + legend.docx]

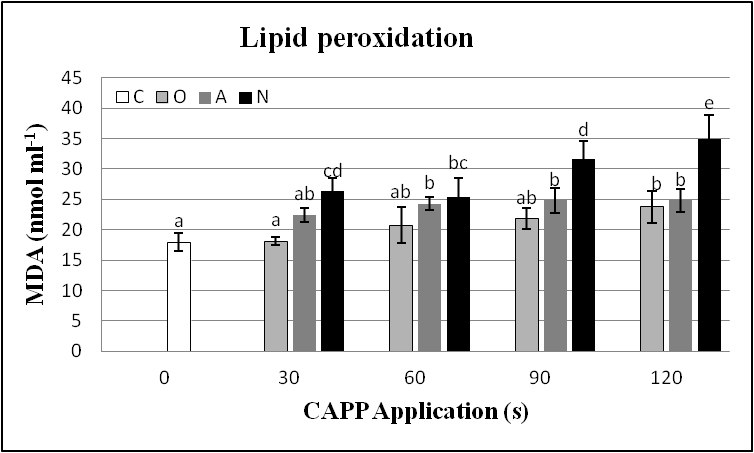


**Figure S1.** Peroxidation of lipids in 3-day-old pea seedlings after CAPP treatment. Variants: C—control/untreated soybean seeds; O30, O60, O90, O120—soybean seeds treated with plasma generated in oxygen atmosphere for 30, 60, 90 or 120 s; A30, A60, A90, A120—soybean seeds treated with plasma generated in ambient air for 30, 60, 90 or 120 s; N30, N60, N90, N120—soybean seeds treated with plasma generated in nitrogen atmosphere for 30, 60, 90 or 120 s. Different letters indicate significant difference at *p*-value < 0.05, bars are means of three experimental runs (one run represents 50 seeds per variant; three 0.5 g mixed samples were analysed per experimental run and each variant) ± SD according to Tukey’s HSD test.
