# Supplementary figures and images for: Evaluation of the Impact of Cold Atmospheric Pressure Plasma on Soybean Seed Germination
Source: Plants (Basel). 2021 Jan 19;10(1):177. doi: 10.3390/plants10010177 (PMC7833387; doi:10.3390/plants10010177)

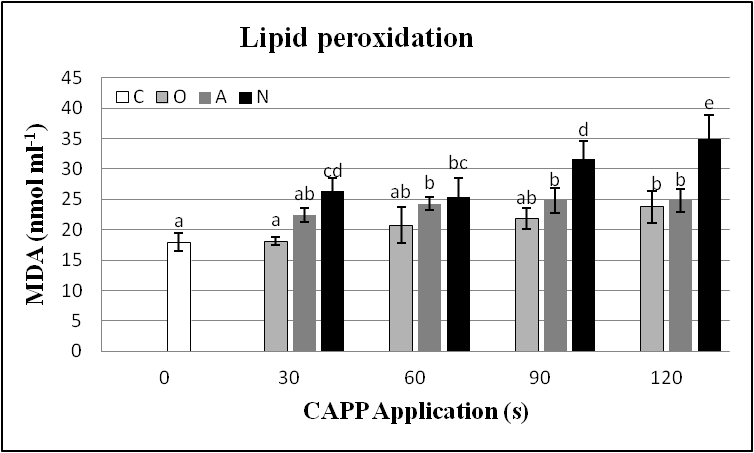

Supplement: Supplementary file 1 [file plants-10-00177-s001.zip › Figure S1.tif]
